# Deletion of sphingosine kinase 2 attenuates cigarette smoke-mediated chronic obstructive pulmonary disease-like symptoms by reducing lung inflammation

**DOI:** 10.17305/bjbms.2022.8034

**Published:** 2023-03-16

**Authors:** Yanhui Chen, Yongrong Zhang, Cheng Rao, Jieyun Huang, Qiong Qing

**Affiliations:** 1 Department of Respiratory Critical Care Medicine, Loudi Central Hospital, Loudi, China

**Keywords:** Sphingosine kinase 2 (SphK2), chronic obstructive pulmonary disease (COPD), cystic fibrosis transmembrane conductance regulator (CFTR), sphingosine-1-phosphate (S1P), pulmonary inflammation

## Abstract

Cigarette smoke (CS) is the leading cause of chronic obstructive pulmonary disease (COPD), which is characterized by chronic bronchial inflammation and emphysema. Growing evidence supports the hypothesis that dysfunctional cystic fibrosis transmembrane conductance regulator (CFTR) is critically involved in the pathogenesis of CS-mediated COPD. However, the underlying mechanism remains unclear. Here, we report that supressed CFTR expression is strongly associated with abnormal phospholipid metabolism and increased pulmonary inflammation. In a CS-exposed mouse model with COPD-like symptoms, we found that pulmonary expression of sphingosine kinase 2 (SphK2) and sphingosine-1-phosphate (S1P) secretion were significantly upregulated. Therefore, we constructed a *SphK2* gene knockout (*SphK2*^−/−^) mouse. After CS exposure for six months, histological lung section staining showed disorganized alveolar structure, increased pulmonary fibrosis, and emphysema-like symptoms in wild-type (WT) mice, which were less pronounced in *SphK2*^−/−^ mice. Further, SphK2 deficiency also decreased CS-induced pulmonary inflammation, which was reflected by a remarkable reduction in pulmonary infiltration of CD45^+^CD11b^+^ neutrophils subpopulation and low levels of IL-6 and IL-33 in bronchial alveolar lavage fluid. However, treatment with S1P receptor agonist suppressed CFTR expression and increased Nf-κB-p65 expression and its nuclear translocation in CS-exposed *SphK2*^−/−^ mice, which also aggravated small airways fibrosis and pulmonary inflammation. In contrast, inhibition of S1P signaling with the S1P receptor analogue FTY720 rescued CFTR expression, suppressed Nf-κB-p65 expression and nuclear translocation, and alleviated pulmonary fibrosis and inflammation after CS exposure. Our results demonstrate that SphK2-mediated S1P production plays a crucial role in the pathogenesis of CS-induced COPD-like disease by impairing CFTR activity and promoting pulmonary inflammation and fibrosis.

## Introduction

Chronic obstructive pulmonary disease (COPD) is the third leading cause of death worldwide. Individuals diagnosed with COPD carry a high risk of lung cancer and poor prognosis after diagnosis and treatment. COPD is characterized by coughing, wheezing, chest tightness, and shortness of breath, and abnormalities in the small airways often result in restriction of airflow in and out of the lungs. Chronic cigarette smoking is one of the most important casual factors contributing to the development of COPD [1, 2]. Cigarette smoke (CS) induces airway inflammation and remodeling; however, how these early changes affect airway obstruction and even emphysema after long-term smoking remains poorly understood.

A growing body of evidence suggests that cystic fibrosis transmembrane conductance regulator (CFTR) plays a critical role in the development of COPD [1, 3]. CFTR dysfunction leads to cystic fibrosis and remodeling of bronchi and small airways, thereby increasing airway resistance and the emergence of pulmonary emphysema [4–6]. Dransfield et al. [2] reported that smokers have substantially lower pulmonary CFTR activity compared with healthy nonsmokers, which was correlated with the disease phenotype of COPD. In addition, reduction of mucus transport in smokers was attributed to the inhibition of CFTR activity and CFTR-dependent fluid transport [7]. In vitro, treatment with CS extract induced cell damage and dysfunction in cultured human bronchial epithelial cells by reducing chloride secretion and increasing mucin synthesis, which was also related to reduced activity of CFTR [8]. Further investigations showed that CS exposure resulted in internalization and retrograde trafficking of CFTR and that the loss of CFTR caused dysfunction and apoptosis of alveolar epithelial and endothelial cells, which led to mucus dehydration and barrier dysfunction [9–12]. Recently, CFTR variants in individuals with a history of smoking were highly associated with COPD and related phenotypes [13]. However, the underlying mechanism for acute effects of CS on CFTR function and its potential role in COPD requires further investigation.

Sphingosine-1-phosphate (S1P), one of the major sphingolipid metabolites synthesized by sphingosine kinases (SphKs), is closely linked to the development of COPD [14]. S1P receptor 1 is highly expressed in lung endothelium and epithelium, where it maintains barrier integrity and function [15, 16]. However, emerging evidence showed that excessive S1P production resulted in bronchial hyperresponsiveness [17]. The hyperactive S1P signaling due to chronic CS inhalation might cause increased airway inflammation, pulmonary cystic fibrosis, and impaired barrier integrity [16]. FTY720 is the S1P analog that antagonizes the S1P effect by competitive binding with the S1P receptor. It regulates pulmonary vascular permeability by preserving the integrity of endothelial barrier [16, 18]. Systemic administration of S1P increased bronchial and small airways responsiveness in both dose- and time-dependent manner [17]. Treatment with TY52156 and JTE-013, the S1P receptor antagonists, effectively reduced lung resistance and improved bronchial function [14]. SphK1 attenuated pulmonary arterial hypertension and bleomycin-induced pulmonary fibrosis [19]. Similarly, deletion of *SphK2* also prevented bacterial-induced pneumonia and lung inflammatory injury [20]. Notably, Malik et al. reported that S1P is a novel regulator of CFTR activity, and increased S1P production attenuated CFTR function by affecting its protein integrity. By contrast, S1P receptor/AMPK inhibition synergistically corrected aberrant CFTR trafficking and barrier function [21]. However, whether CFTR function regulated by the sphingosine kinase 2 (SphK2)/S1P pathway has a potential role in CS-mediated COPD has not been established.

In the present study, we found that SphK2 was upregulated and S1P production increased in the lungs of CS-exposed mice. Therefore, we hypothesized that SphK2 might be critically involved in CFTR dysfunction and COPD development after chronic CS exposure. In a *SphK2* knockout (*SphK2^−/−^*) mouse model, we revealed that CS-induced pulmonary inflammation and fibrosis were significantly decreased, and SphK2 deficiency rescued CFTR activity and preserved pulmonary function by suppressing the S1P secretion. Our findings demonstrate a key role of SphK2 in the pathology of CS-mediated COPD by impairing CFTR function, which represents an effective therapeutic strategy against COPD through inhibition of S1P signaling.

## Materials and methods

### Generation and breeding of *SphK2* knockout (*SphK2*^−/−^) mice

*SphK2*^−/−^ mice, in which exons 4~6 of the *SphK2* gene were deleted using the CRISPR-Cas9 strategy, were developed following the gene knockout protocol provided by Cyagen Biomodels (Guangzhou, China). These mice were kindly obtained from Dr. Tony Wang (Fudan University). Male homozygous knockout (*SphK2*^−/−^) mice and the wild-type (WT) control mice used in the experiments were obtained by breeding heterozygous (*SphK2*^+/−^) mice for more than six generations. Genotyping was performed by polymerase chain reaction (PCR) by extracting tail-vein DNA from 2-week-old mice to confirm the deletion of exons 4~6 in the *SphK2* gene. In brief, mouse tail-vein was cut 0.5~1-cm long, ground into powder in liquid nitrogen, and transferred into a 1.5-mL centrifuge tube. Then 200 L of lysis buffer and 5 L of proteinase K (Yeasen Biotechnology, Co., Ltd.) were added to the tube, mixed with gentle vortex, and incubated at 55 ^∘^C for 3 h. After the tube was brought back to room temperature, 200 L of PL buffer and 200 L of BD buffer (Yeasen Biotechnology, Co., Ltd.) were subsequently added to the tube with vigorous shaking. The solution was separated after centrifuge at 12000 rpm for 5 min. DNA samples were carefully extracted from the bottom layer phase of the solution. After purification and column elution, the DNA samples were subjected to PCR procedures. PCR was performed using the following primer sets: forward primer: 5’-TATGGGTGTTGCCATGTGTCC-3’; and reverse primer: 5’-AGAGCAGGTCCAGAGGATGG-3’. In the WT mice, these set primers were used to amplify a fragment of approximately 1500 bp, and in case of the *SphK2*^−/−^ mice, a fragment of approximately 550 bp was amplified. *SphK2*^−/−^ mice are physiologically normal like WT mice, but smaller than age-matched normal WT mice. All mice used in the study were treated with care in accordance with protocols established by the University of South China Animal Care and Use Committee.

### Cigarette smoke exposure

Eight-week-old WT and *SphK2*^−/−^ mice were raised in our animal facility and exposed to mixed CS in a closed environment for one hour each day. Briefly, mice were placed in an iron chamber which was connected to a Smoke Generator (Yuyan Instruments, Co., Ltd.) by the ventilation pipe. Smoke contained a tar content of 12 mg per cigarette, and the nicotine content was 2.5 mg per cigarette, which was conducted by intermittently forcing air (2 L/min) after burning cigarettes. In total, 5 mice were exposed to smoke from 5 cigarettes (1 h) each day for 5 days per week over a period of 6 months. Mice (*n* ═ 5) in the control group were exposed to filtered air instead of CS. Mice (*n* ═ 5) in the treatment group received FTY720 (5 mg/kg/day, cat #S5002, Selleck) via intraperitoneal injection or CYM-5442 (2 mg/kg/day, cat #S2878, Selleck) via intragastric administration.

### Lung tissue fixation and immunohistochemical analysis

At the end of the experiments, mice were sacrificed after inhalation of isoflurane (4%), and lung tissue was collected and fixed by tracheal instillation of 4% PFA and embedded in paraffin for immunohistochemical staining. Each tissue section (5 µm) was cut and stained with Masson’s trichrome, following the standardized manufacturer’s protocols after deparaffinization. The stained blue areas around the airways were identified as subepithelial collagen deposits. The percentages of Masson-stained areas in relation to the total epithelial area and alveolar chord length were calculated using Image J software. The mean alveolar chord length was calculated as the average of the horizontal and vertical distances between the alveolar walls. In addition, fixed sections were immunohistochemically stained with an anti-alpha smooth muscle (α-SMA) antibody (1:200, AF1507, Beyotime Biotechnology, Shanghai, China).

### Bronchial alveolar lavage fluid and cell collection

Bronchial alveolar lavage fluid **(**BALF) was collected as previously described [22]. Briefly, mice were euthanized with 4% isoflurane inhalation. The tracheas were cannulated, and the lungs were lavaged in situ with 500 µL increments of ice-cold PBS 5 to 6 times. The average liquid recovery after lavage was greater than 60%. Supernatant fluid was aliquoted into small samples and frozen at –80 ^∘^C for ELISA of cytokines. The cell mass was resuspended in 500 µL PBS, isolated by centrifugation and processed for further flow cytometry.

### RNA extraction and reverse transcription-polymerase chain reaction

At the designated intervals after CS exposure, mice were anesthetized and sacrificed, followed by rapid separation of the lung tissue. After washing for ice-cold 1×PBS twice to remove residual blood, the total RNA from the tissue was extracted using TRIzol^^®^^ reagent (Thermo Fisher Scientific, Inc.), following the manufacturer’s instructions. RNA quality was analyzed under a UV spectrophotometer, followed by reverse transcription of the RNA to cDNA using the reverse transcription kit (D7168L; Beyotime Institute of Biotechnology). Using the cDNA as a template, the transcriptional level of *SphK2* gene in lung tissue was measured by RT-PCR under the following conditions: an preliminary denaturation phase at 94 ^∘^C for 2 min, followed by 35 cycles of denaturation at 94 ^∘^C for 30 s, annealing at 55 ^∘^C for 30 s and extension at 72 ^∘^C for 30 s, and a final extension at 72 ^∘^C for 5 min. The *SphK2* gene primers were: forward, 5’TGGCTGCTTCATTGTTCTTG3’; and reverse, 5’TTCTCTACCTTTCCGCCAGA3’. PCR products were resolved in 1.2% agarose gel and electrophoresed for 20 min. The blots were visualized under a UV spectrophotometer and quantified using Quantity One 1-D analysis software (Bio-Rad, Hercules, CA, USA).

### Western blotting analysis

Lung tissues were washed with ice-cold 1×PBS and then homogenized and lysed in 150 µL of protein lysis (RIPA) buffer containing 1% PMSF (ST506, Beyotime Biotechnology, Shanghai, China). Protein lysates were harvested and electrophoresed on a 10% Bis–Tris gel (Invitrogen). Following transfer to PVDF membrane (Millipore), the protein blots were blocked with 5% BSA for 1 h and incubated overnight with the primary antibodies at 4 ^∘^C. The primary antibodies specific for SphK2 (A01382-1, Boster Biol. Tec.), CFTR (ab181782, Abcam, Cambridge, UK), and Nf-κB-p65 (#3036, Cell Signaling Technology, Beverly, MA, USA) were used. Subsequently, the blots were washed with TBST 3 times and then incubated with the HRP-labeled secondary antibodies for 30 min at room temperature. The immunoreactive bands were visualized by Enhanced ECL Chemiluminescent Substrate Kit (Thermo Scientific, USA).

### Nf-κB activity assay

Paraffin sections of lung tissues were dewaxed with xylene, hydrated with gradient ethanol, and antigen retrieved with a solution of citrate buffer (pH 7.0–8.0) in a microwave oven. After washing with PBS for 3 times, the sections were treated with 0.2% Triton X-100 for 20 min at room temperature. Then the sections were incubated with 3% BSA for 1 h, and further incubated with anti-phospho-Nf-κB-p65 antibody (SN371, Beyotime BiotechShanghai, China) and anti-Elastin (ab21610, abcam, Cambridge, UK) at 4 ^∘^C overnight. After washing with PBS for 3 times, the sections were incubated with appropriate fluorescent secondary antibody and Dapi for 2 h and 5 min, respectively. After washing with PBS for 3 times, the slides were mounted with glycerin buffer and observed using a fluorescence microscope.

### ELISA analysis

The levels of IL-6, IL-33, and S1P in the collected BALF of lung samples were measured using commercial ELISA kits (PI326 and PI627, Beyotime Biotechnology, Shanghai, China; K-1900; Echelon Bioscience, USA) following the manufacturer’s instructions.

### Fluorescence-activated cell sorting analysis

Cells collected from BALF were washed with red blood cell lysis buffer (pH 7.4) to remove the residual red blood cells and were counted with Trypan blue. Approximately 2×10^5^ cells of each sample were fixed immediately in 1.5% paraformaldehyde for 10 min at 4 ^∘^C. The cells were then washed with 1 × PBS and stained with anti-CD45-FITC antibody (ab210184) and anti-CD11b-PE antibody (ab25175). After staining, the cells were washed twice with 1 × PBS and subjected to fluorescence-activated cell sorting analysis. Flow cytometry data were analyzed using the software FlowJo7.6 (Tree Star, Inc. Ashland, OR, USA).

### Ethical statement

All mice used in this study were housed under standard laboratory and ethical conditions with reversed light-dark cycle (12-h light/12-h dark) and with food and water available ad libitum. All protocols conformed to Declaration of Helsinki principles and in accordance with protocols established by the University of South China Animal Care and Use Committee.

### Statistical analysis

Data were expressed as mean ± standard error of the mean (SEM). Statistical analysis was performed with Prism GraphPad8.0 (GraphPad Software Inc., San Diego, CA, USA). Two-tailed Student’s t-test, one-way ANOVA, or two-way ANOVA, followed by Tukey’s post-hoc analyses were used to compare different groups. A *P* value <0.05 was considered statistically significant. Randomization and blinding strategy were used whenever possible.

**Figure 1.**
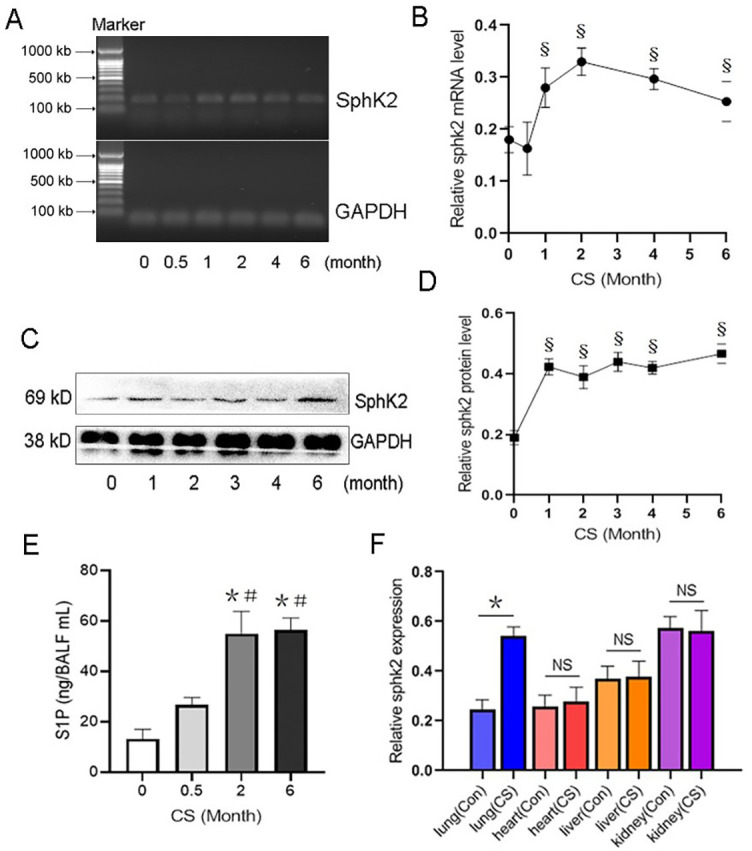
**Increased SphK2 caused by chronic CS exposure in the lung tissue of WT mice.** (A) Mice were subjected to CS exposure 5 days a week for 6 months; the *SphK2* mRNA levels in the lung tissues were measured and (B) quantified by RT-PCR at indicated times (0, 0.5, 1, 2, 4 and 6 months) after CS exposure; (C) The SphK2 protein levels in the lung tissue were measured and (D) quantified by western blot at indicated times (0, 1, 2, 3, 4 and 6 months) after CS exposure (*n* ═ 4, ^§^*P* < 0.05, vs 0-month group); (E) The S1P levels in the collected BALF were measured by ELISA (*n* ═ 4, ^*^P < 0.05, vs non-CS exposure; *P* < 0.05, vs CS exposure for 30 days); (F) Quantification of *SphK2* mRNA levels in lung, heart, liver, and kidney of mice exposed to air or CS for six months (*n* ═ 5, ^**^P < 0.05, vs control group (Con) of lung, heart, liver, or kidney tissue). SphK2: Sphingosine kinase 2; CS: Cigarette smoke; WT: Wild-type; RT-PCR: Real-time polymerase chain reaction; BALF: Bronchoalveolar lavage fluid; GADPH: Glyceraldehyde-3-phosphate dehydrogenase.

## Results

### *SphK2* is upregulated in lung tissue of WT mice after CS exposure

Previous studies reported that the endogenous levels of S1P were highly associated with the development of COPD [23, 24]. Here, we investigated the role of SphK2, which converts sphingosine to active S1P in COPD pathogenesis. During CS exposure, the levels of *SphK2* mRNA were rapidly upregulated in the lung tissue of WT mice within the first three months, followed by a peak and subsequent decline. However, the *SphK2* levels remained higher than baseline after CS exposure for six months (Figure 1A and 1B). Consistently, the protein levels of SphK2 also increased continuously and remained elevated during the CS exposure (Figure 1C and 1D). After six months of CS exposure, the levels of S1P in BALF were significantly higher than in BALF of control mice or mice exposed to CS for half a month (Figure 1E), indicating that upregulation of SphK2 promoted a significant increase in S1P secretion in the lungs of mice after long-term CS exposure. Considering that smoking-induced organ injury may not be limited to the lungs in exposed to CS, we further analyzed the transcriptional changes of *SphK2* expression in other organs in CS-exposed mice. CS exposure for 6 months resulted in a 2.2-fold increase *in SphK2* mRNA specifically in lung tissue (Figure 1F), which was not observed in heart, liver, and kidney, indicating that the upregulation of SphK2 is highly associated with CS-induced lung injury.

**Figure 2.**
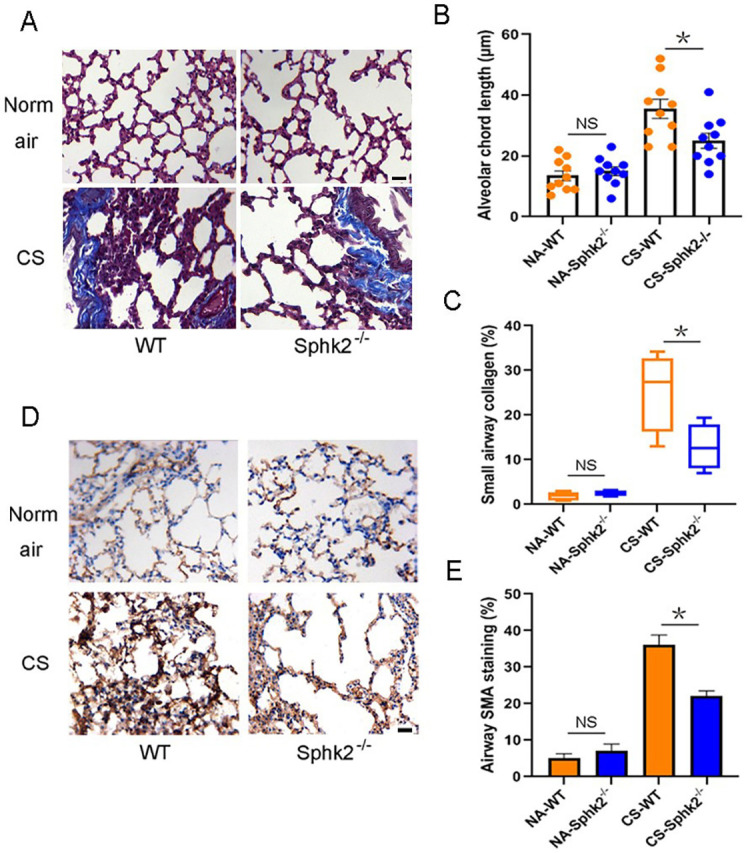
**CS-induced pulmonary fibrosis was alleviated in *SphK2*^−/−^ mice.** WT and *SphK2*^−/−^ mice were both subjected to air or CS exposure 5 days a week for 6 months. (A) Masson’s trichrome staining reveals the alveolar structure (red) and collagen deposition (blue) around the small airways of lung tissues of WT and *SphK2*^−/−^ mice; (B) Morphometric changes in alveolar walls were measured as mean alveolar chord length on HE-stained lung sections by computer-assisted image analysis (*n* ═ 10, ^*^P < 0.05, vs CS-exposed WT mice; scale bar ═ 50 µm); (C) Quantification (%) of small airways collagen deposition (*n* ═ 4 mice per group; ^*^P < 0.05, vs CS-exposed WT mice); (D) Immunohistochemical staining shows anti-α-SMA staining of alveolar walls in WT and *SphK2*^−/−^ mice exposed to normal air or CS; scale bar ═ 50 µm; (E) Quantification (%) of anti-α-SMA-positive cells around small airways (*n* ═ 4, ^*^P < 0.05, vs CS-exposed WT mice). SphK2: Sphingosine kinase 2; CS: Cigarette smoke; WT: Wild-type; RT-PCR: Real-time polymerase chain reaction; BALF: Bronchoalveolar lavage fluid; NA: Normal air; α-SMA: α-smooth muscle actin.

**Figure 3.**
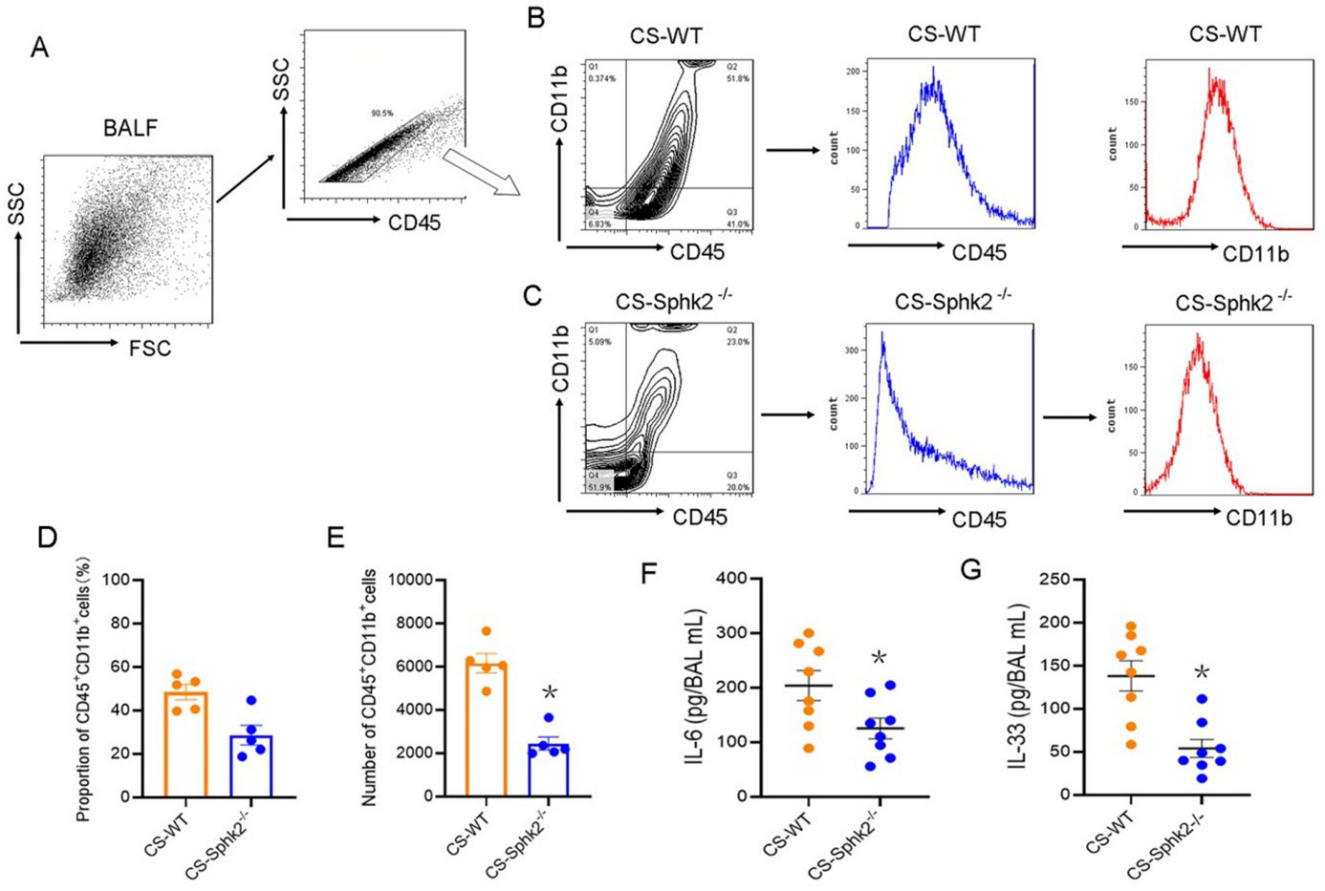
**CS-induced pulmonary inflammation was attenuated in *SphK2*^−/−^ mice.** WT and *SphK2*^−/−^ mice were both subjected to air or CS exposure 5 days a week for 6 months. The BALF was collected from mice of both genotypes and analyzed by flow cytometry. (A) BALF cells were sorted by FACS with anti-GFP antibody, followed by gating with anti-CD45 antibody; (B) Representative cytoflow sorting images show the proportion of CD45^+^CD11b^+^ subsets, and representative histograms indicate the normal distribution frequency of anti-CD45 (blue line) and anti-CD11b (red line) in BALF obtained from CS-exposed WT and (C) *SphK2*^−/−^ mice based on mean fluorescence intensity; (D-E) Quantification of the proportion and the number of CD45^+^CD11b^+^ subsets (*n* ═ 5, ^*^P < 0.05 vs CS-exposed WT mice); (F-G) The levels of IL-6 and IL-33 in BALF were determined by ELISA (*n* ═ 8, ^*^P < 0.01 vs CS-exposed WT mice). SphK2: Sphingosine kinase 2; CS: Cigarette smoke; WT: Wild-type; BALF: Bronchoalveolar lavage fluid; FACS: Fluorescence-activated cell sorting.

### Genetic deletion of *SphK2* attenuates pulmonary fibrosis and alleviates CS-induced COPD-like disease

**Figure S1.**
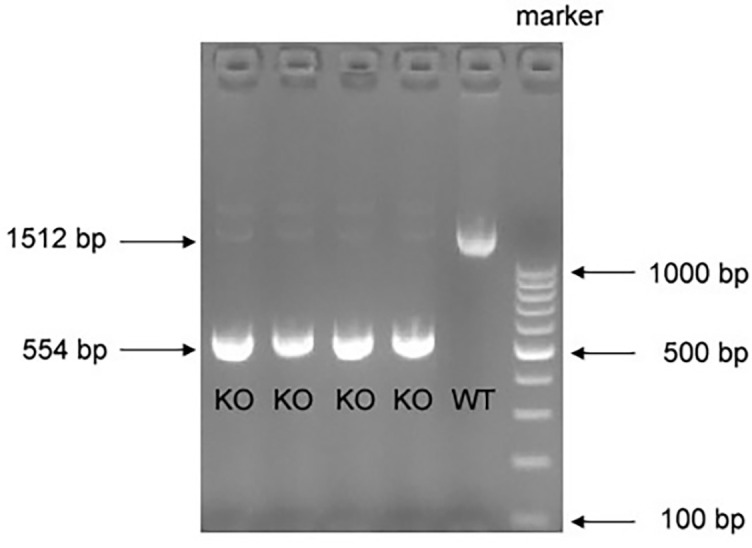
**PCR analysis showing the genotyping of *SphK2***^−/−^**mice.** SphK2: Sphingosine kinase 2; WT: Wild-type; KO: Knockout.

**Figure S2.**
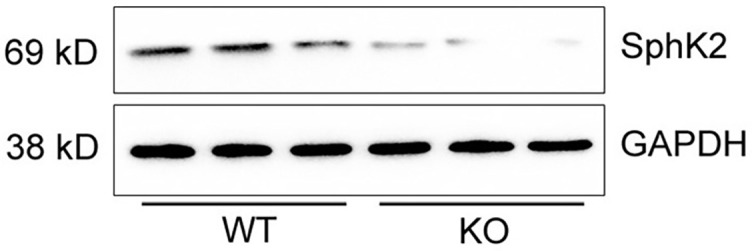
*****SphK2*** knockdown in lung tissue of *SphK2***^−/−^**mice was confirmed by western blotting.** SphK2: Sphingosine kinase 2; WT: Wild-type; GADPH: Glyceraldehyde-3-phosphate dehydrogenase; KO: Knockout.

To determine the effect of SphK2 on CS-induced pulmonary injury, we generated *SphK2* gene knockout (*SphK2*^−/−^) mice and backcrossed with a C57 background (Figure S1 and S2). *SphK2*^−/−^ mice displayed lung development and normal phenotype at the baseline as shown by normal pulmonary interstitial, alveolar, and bronchial epithelium. Exposure to CS for six months led to alveolar wall defects and destruction in WT mice, but less frequently in *SphK2*^−/−^ mice (Figure 2A). The mean chord length is the average of the horizontal and vertical distances between the alveolar walls, therefore it is commonly used for the analysis of airspace enlargement [35, 36]. WT mice exposed to CS for six months displayed markedly increased airspace in the lung relative to that seen in normal air-treated controls (Figure 2B). By contrast, we observed less airspace enlargement and attenuated small airways fibrosis in CS-exposed *SphK2*^–/–^ mouse lungs (Figure 2B and 2C). In addition to airspace enlargement, increased collagen production was also observed in lungs after chronic CS exposure. In addition to attenuated fibrosis, we found that the accumulation of α-SMA positive cells around the small airways was significantly reduced in *SphK2*^−/−^ mice compared with those in WT mice (Figure 2D and 2E), suggesting that SphK2 deficiency attenuated the pathological fibrotic and emphysema manifestations in mice with chronic CS exposure.

### CS-induced pulmonary inflammation is attenuated in *SphK2*^−/−^ mice

An abundance of evidence suggests the role of inflammation in the development and progression of CS-induced COPD [1, 22]. Therefore, we investigated whether SphK2 deficiency attenuated pulmonary inflammatory response following CS exposure. CS-induced infiltration of inflammatory cells in collected BALF, which was analyzed by flow cytometry. As shown in Figure 3A–3C, more than 90% of leukocytes were selected by gating with an anti-CD45 antibody. The proportions of CD45^+^CD11b^+^ subsets in leukocytes were analyzed both in CS-exposed WT mice and *SphK2*^−/−^ mice. The results showed that the CD45^+^CD11b^+^ subsets in BALF of *SphK2*^−/−^ mice (23.0%) were significantly decreased when compared with those of WT mice (51.8%). Consistent with the higher frequencies of CD45-expressing and CD11b-expressing cells observed in WT mice, the cell number and the proportion of CD45^+^CD11b^+^ subpopulations in WT mice were both significantly higher than those in *SphK2*^−/−^ mice (Figure 3D and 3E). Importantly, we also found that the secretion of pleiotropic cytokines such as IL-6 and IL-33, which are predominantly observed in lung tissue and lead to airway inflammation [22], was significantly reduced in the BALF of *SphK2*^−/−^ mice compared with the levels in the BALF of WT mice (Figure 3F and 3G), strongly suggesting a critical role of SphK2 in inducing pulmonary inflammation after chronic CS exposure.

**Figure 4.**
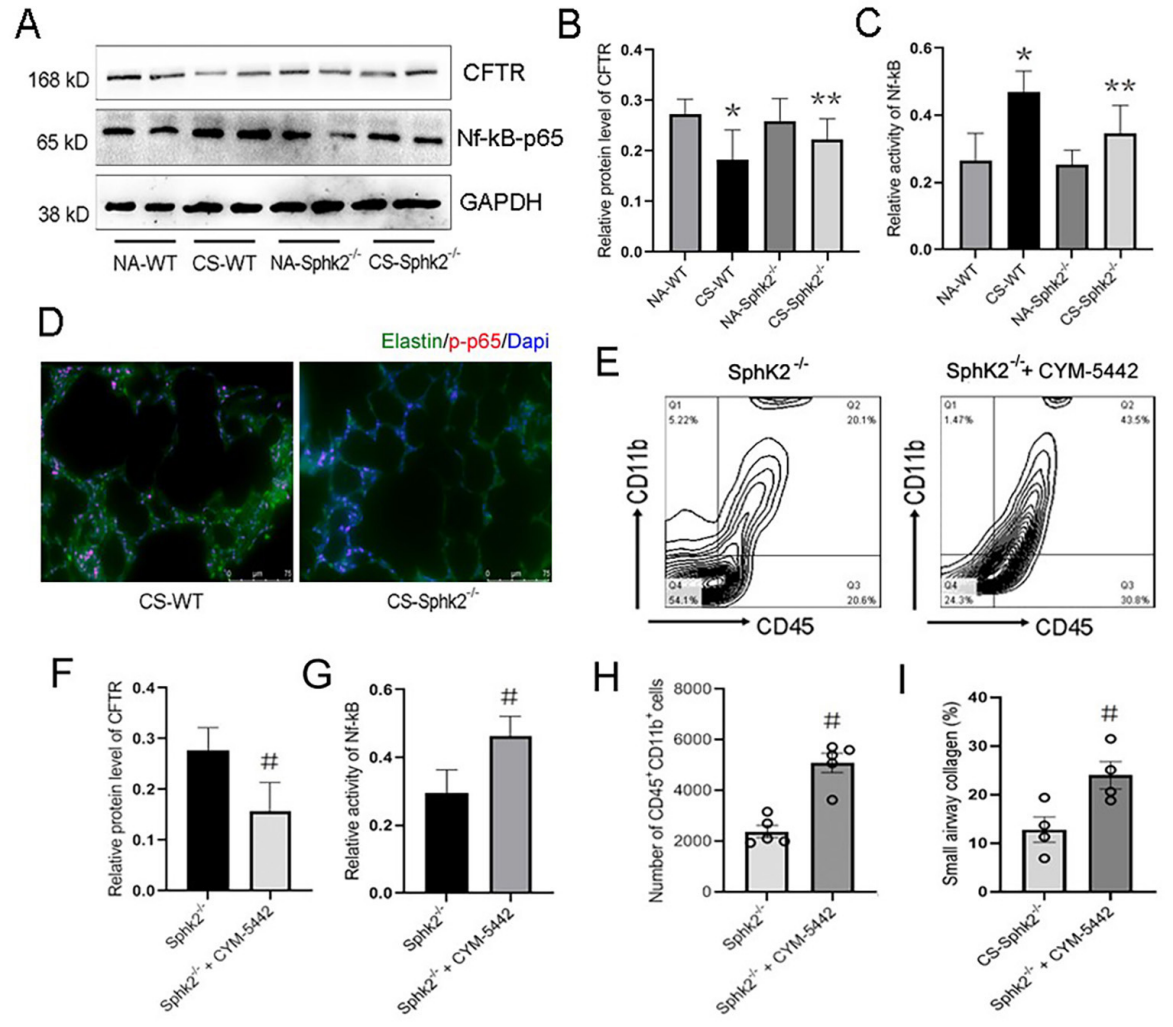
**Reduced CFTR expression and attenuation of pulmonary inflammation and fibrosis in CS-exposed **SphK2**^−/−^ mice.** (A) Western blotting analysis showed that the protein expression of CFTR, phospho-Nf-κB-p65, and GAPDH in lung tissues of WT mice and ***SphK2***^−/−^ mice under normal air or CS exposure; (B-C) Quantification of the relative protein expression of CFTR and phospho-Nf-κB-p65 (*n* ═ 4, ^*^P < 0.05, vs NA-exposed WT mice; ^**^P < 0.05, vs CS-exposed WT mice); (D) Immunofluorescence staining of elastin (green)/phospho-Nf-κB-p65 (red)/Dapi (blue) of lung tissue sections from CS-exposed WT mice and *SphK2*^−/−^ mice; (E) Representative cytoflow sorting images show the proportion of CD45^+^CD11b^+^ cells in BALF of *SphK2*^−/−^ mice treated with or without S1P_1_ agonist; (F-G) Quantification of the relative protein expression of CFTR and phospho-Nf-κB-p65 in lung tissues of *SphK2*^−/−^ mice treated with or without CYM-5442. (*n* ═ 4, ^#^*P* < 0.05, vs CS-exposed *SphK2*^−/−^ mice); (H) Quantification of the number of CD45^+^ subsets and CD45^+^CD11b^+^ subsets (*n* ═ 5, ^#^*P* < 0.05, vs CS-exposed *SphK2*^−/−^ mice); (I) Quantification (%) of small airways collagen deposition (*n* ═ 4, ^*^P < 0.05, vs CS-exposed *SphK2*^−/−^ mice). SphK2: Sphingosine kinase 2; CS: Cigarette smoke; WT: Wild-type; BALF: Bronchoalveolar lavage fluid; GADPH: Glyceraldehyde-3-phosphate dehydrogenase; CFTR: Cystic fibrosis transmembrane conductance regulator.

**Figure 5.**
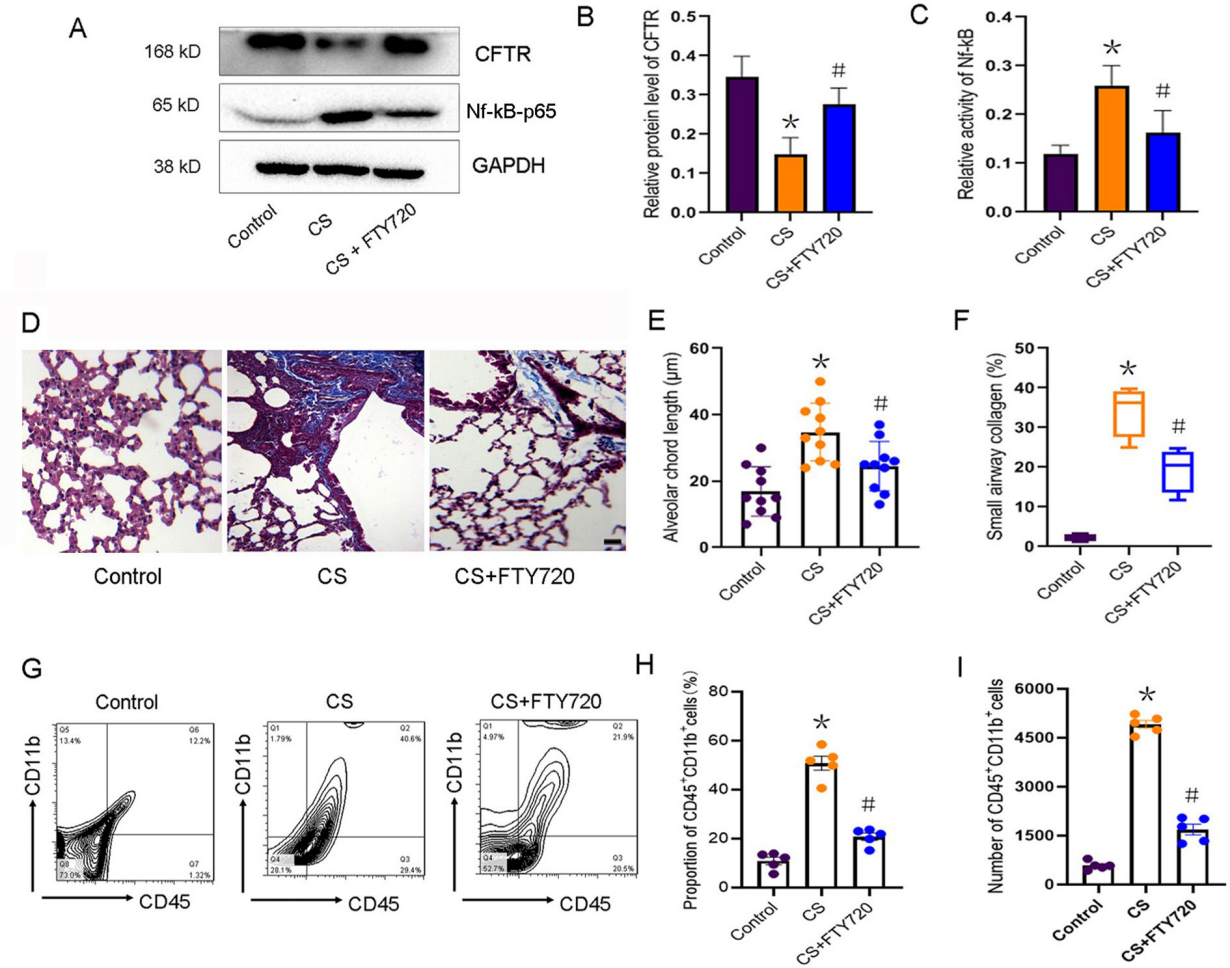
**FTY720 treatment rescued CFTR function and attenuated pulmonary inflammation and fibrosis in CS-exposed mice.** WT mice treated with or without FTY720 were both exposed to CS 5 days a week for 6 months. (A) Western blotting analysis reveals the protein levels of CFTR and phospho-Nf-κB-p65 in lung tissues of mice; (B-C) Quantification of the relative protein expression of CFTR and phospho-Nf-κB-p65 in lung tissues of mice (*n* ═ 4, ^*^P < 0.05, vs control mice; ^#^*P* < 0.05, vs CS-exposed WT mice); (D) Masson’s trichrome staining shows the alveolar structure (red) and collagen deposition (blue) in lung tissues of mice; (E) The alveolar chord length was measured on HE-stained lung sections (*n* ═ 10, ^*^P < 0.05, vs control mice; ^#^*P* < 0.05, vs CS-exposed WT mice); (F) Quantification (%) of small airway collagen deposition (*n* ═ 4, ^*^P < 0.05, vs control mice; ^#^*P* < 0.05, vs CS-exposed WT mice); (G) Representative cytoflow sorting images show the proportion of CD45^+^CD11b^+^ subset in BALF of mice; (H-I) Quantification of the proportions and the number of CD45^+^CD11b^+^ subsets (*n* ═ 5, ^*^P < 0.05, vs control mice; ^#^*P* < 0.05, vs CS-exposed WT mice). SphK2: Sphingosine kinase 2; CS: Cigarette smoke; WT: Wild-type; BALF: Bronchoalveolar lavage fluid; GADPH: Glyceraldehyde-3-phosphate dehydrogenase; CFTR: Cystic fibrosis transmembrane conductance regulator.

### SphK2 deficiency preserved CFTR function, which was highly associated with attenuated pulmonary fibrosis and inflammation after CS exposure

To investigate the mechanism underlying SphK2-mediated pulmonary emphysema, remodeling, and inflammation, we analyzed the effect of SphK2 deficiency on protein expression of CFTR. The absence or dysfunction of CFTR in the alveolar epithelium lining caused decreased Cl^-^ secretion and increased Na^+^ reabsorption, suggesting that CS-mediated COPD might be due, at least in part, to reduced CFTR activity and related cystic fibrosis and pulmonary inflammation [2, 25]. One recent finding showed that S1P is a novel regulator of CFTR activity [16]. Therefore, we determined the CFTR expression in the lung tissue of both WT and *SphK2*^−/−^ mice after CS exposure. Following CS exposure for two months, the protein expression of CFTR was significantly decreased in the lung tissue of WT mice (Figure 4A). Although CFTR expression and activity were also reduced in *SphK2*^−/−^ mice compared with those not exposed to CS, it was largely improved when compared with CS-exposed WT mice (Figure 4A and 4B). The decrease of CFTR was accompanied by a significant increase in Nf-κB-p65 activity indicated by the phosphorylation of p65 in the lung tissue of WT mice, which was also reversed by SphK2 deficiency (Figure 4A and 4C). Nf-κB activation promotes dissociation of p50/p65 heterodimers, and phosphorylation of p65 facilitates its nuclear translocation and increased nucleotide-binding and transcriptional activity. Therefore, we further determined the DNA-binding p65 expression in lung tissue of CS-exposed mice. By immunofluorescence staining, we observed that the anti-phospho-p65 fluorescence intensity overlapping with Dapi pre-stained nuclei was significantly attenuated in *SphK2*^−/−^ mice compared with WT mice (Figure 4D), indicating that CS-induced p65 activation and nuclear translocation were markedly suppressed by deletion of *SphK2* in lung tissue. These data indicated that SphK2 deficiency might alleviate CS-induced pulmonary inflammation by rescuing the CFTR function and suppression of Nf-κB activity.

Recently, S1P was reported as a novel regulator of CFTR activity [16]. We therefore pretreated *SphK2*^−/−^ mice with CYM-5442, a S1P_1_ agonist (2 mg/kg/day) during the CS exposure. Compared with the *SphK2*^−/−^ mice without CYM-5442 treatment, a significant reduction in CFTR expression and an increase in Nf-κB activity were observed in the lung tissue of *SphK2*^−/−^ mice (Figure 4F and 4G). Meanwhile, CYM-5442 also enhanced small airways fibrosis and pulmonary inflammation as reflected by increased infiltration of CD45^+^CD11b^+^ monocytes in the BALF (Figure 4E, 4H, and 4I). These data suggested that SphK2-mediated S1P production aggravated CS-induced experimental COPD-like disease by inhibiting pulmonary epithelial CFTR activity.

### S1P signaling inhibition restored CFTR activity and alleviated pulmonary fibrosis and inflammation after chronic CS exposure

**Figure S3.**
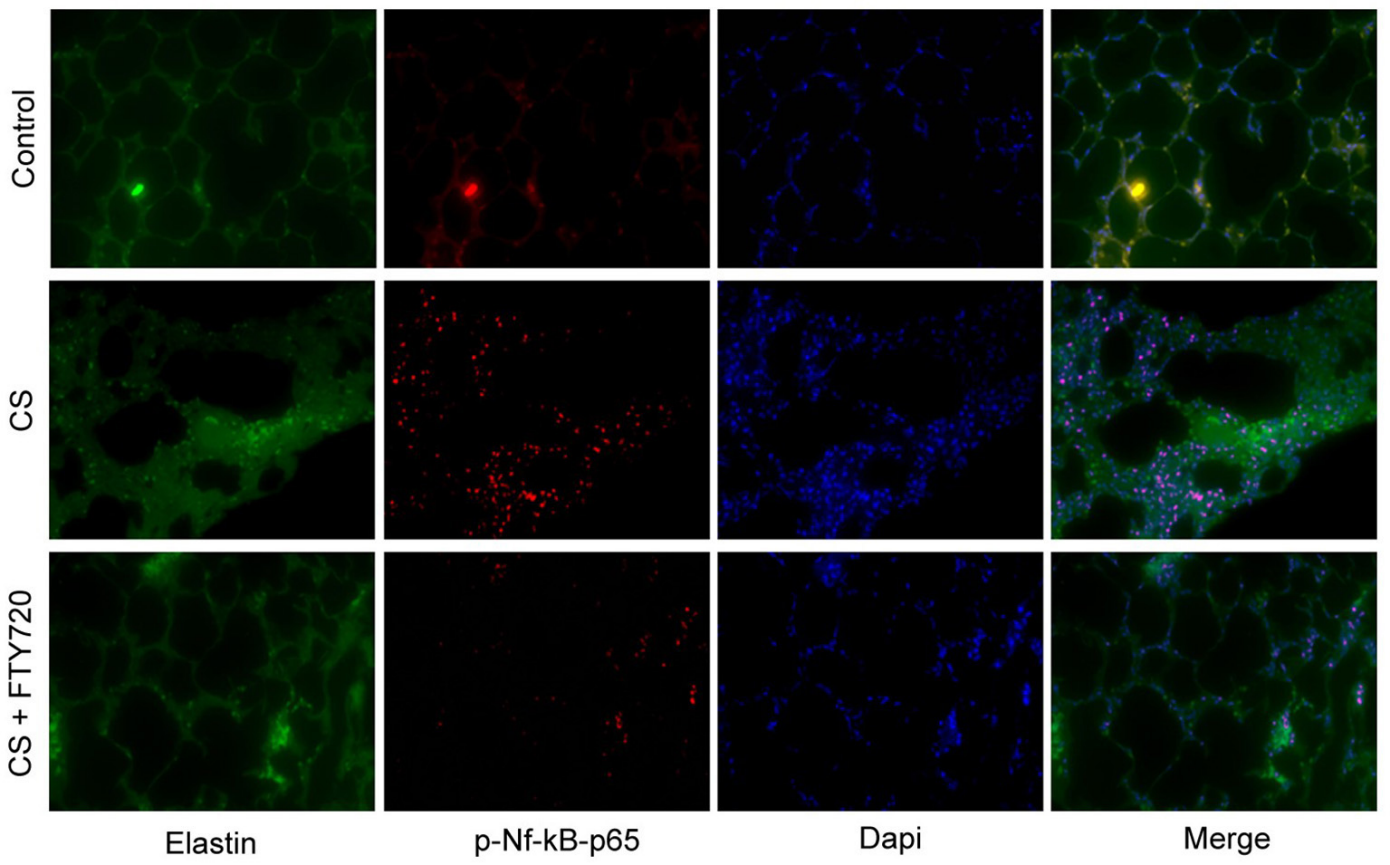
**NF-κB DNA binding activity was determined by immunofluorescence staining.** Represent images showing elastin (green)/phospho-Nf-κB-p65 (red)/Dapi (blue) of lung tissue sections from control mice, CS-exposed WT mice and CS-induced WT mice with FTY720 treatment. SphK2: Sphingosine kinase 2; WT: Wild-type; CS: Cigarette smoke.

To further establish the key role of S1P in regulating CFTR activity and related COPD development, we blocked the S1P signaling by pretreating the WT mice with FTY720 (10 mg/kg/day), which belongs to a new class of immunosuppressants that inhibit S1P signaling by competitively binding with S1P receptors. After CS exposure for six months, FTY720 treatment significantly increased the expression of CFTR and reduced the phospho-Nf-κB-p65 expression (Figure 5A–5C). Increased nuclear DNA-binding p65 in lung tissue after CS exposure was also significantly reduced by the treatment of FTY720 (Figure S3), suggesting a key role of S1P in triggering CS-mediated pulmonary inflammation. After six months of CS exposure, the inhibition of S1P by FTY720 effectively prevented alveolar enlargement (Figure 5E) and reduced lung fibrosis (Figure 5D and 5F), which was reflected by a significant decrease in small airways collagen formation. Further, FTY720 treatment markedly decreased the proportion and cell number of CD45^+^CD11b^+^ subpopulations in lung tissue of CS-exposed mice (Figure 5G–5I). These data indicated that S1P signaling plays a critical role in CS-induced COPD development, suggesting that targeting SphK2, which is responsible for S1P section post-CS exposure, is an effective therapeutic strategy against COPD.

## Discussion

S1P, the major product of sphingolipid metabolism, plays an important role in the pathogenesis of lung fibrosis [25–28]. S1P is highly expressed in pulmonary epithelial and endothelial cells. Inhibition of S1P biosynthesis attenuates pulmonary inflammation, airway hyperresponsiveness, and allergen-induced asthma-like features, while the administration of S1P worsens airway inflammation and allergic bronchial asthma [29, 30]. Additionally, the elevated S1P level is strongly associated with bronchial and small airways fibrosis in COPD [26, 31, 32]. Recently, De Cunto et al. addressed a big contribution of S1P in airways pathology and COPD development in a mouse CS model, they demonstrated the therapeutic potential of S1P inhibitors in controlling emphysema-related airways hyperresponsiveness in smokers with both asthma and COPD [14]. Consistently, in the present study, we revealed that S1P production was significantly reduced in the lung tissue of *SphK2*^−/−^ mice after CS exposure. Pulmonary inflammation and small airways fibrosis were also markedly decreased after CS exposure. In line with the previous studies [14, 23], we found that CS-induced experimental COPD was improved in *SphK2*^−/−^ mice, at least in part, due to the failure of S1P production. Further, we defined a possible link between increased S1P levels and pulmonary inflammation and airways fibrosis in vivo, which suggested a potential mechanism for CS exposure-induced pulmonary dysfunction and remodeling.

Bronchial and small airways fibrosis is often the most typical manifestation of COPD, which usually leads to air wall thickness, emphysema and difficulty breathing. Previous studies showed that SphK2, catalyzing the conversion of sphingosine to S1P, played a critical role in renal fibrosis [33, 34]. However, the role of SphK2 in CS-induced COPD remained unclear. Here, we found that SphK2 was upregulated in lung tissue after CS exposure in a time-dependent manner; however, SphK2 remained elevated during the development of experimental COPD. In line with the elevation of SphK2, S1P production and pulmonary fibrosis were also significantly increased. Therefore, the deletion of *SphK2* is expected to improve CS-induced small airway remodeling and pulmonary dysfunction. Indeed, decreased α-SMA positive cells around the small airways and less severe emphysema were observed in *SphK2*^−/−^ mice. Collagen deposition is well-known to promote epithelial-mesenchymal transition and subsequent thickening of small airways [37–39], which might be the primary cause of COPD [40, 41]. Our results suggested that deletion of *SphK2* also reduced epithelial-mesenchymal transition in CS-induced lung tissue. Whether the epithelial-mesenchymal transition effect is regulated by Sphk2/S1P pathway or is the result of Sphk2-mediated pulmonary inflammation requires further research in the future.

To gain insight into the mechanism underlying SphK2-mediated pulmonary fibrosis under chronic CS exposure, we investigated the CFTR expression in lung tissues from both WT and *SphK2*^−/−^ mice in response to CS exposure. Previous studies revealed that reduced lung CFTR expression was closely associated with ion transport defects which might contribute to the pathological changes seen in COPD [1, 5, 24]. CFTR is involved in cAMP-dependent fluid transport from the distal air spaces of the lung [11]. CS exposure induced internalization and insolubility of CFTR and resulted in dehydration of airways surface fluid, which promoted mucus stasis, failure of mucus clearance, and the development of chronic bronchitis [10]. In our study, either deletion of *SphK2* or suppression of S1P rescued CFTR expression in CS-exposed mice. However, Wong, FH. et al. in their study reported that CFTR-mediated secretion during acute CS exposure initially protected the airways epithelium, but prolonged CS exposure still led to CFTR functional repression and reduced the efficacy of drugs for cystic fibrosis. Therefore, CFTR plays an important role in maintaining the homeostasis of bronchial epithelium and lowering the airways hyperresponsiveness and dysfunction [42]. Increased CFTR expression on pulmonary epithelial and endothelial cells controls Cl^-^ secretion and Na^+^ reabsorption, which facilitates E-cadherin expression and barrier integrity of small airways [12, 39]. Roflumilast functioned as a CFTR activator, reversed CFTR dysfunction and increased intestinal fluid in mice in a CS exposure-dependent manner, and benefited COPD patients with bronchitis [5, 43].

Although multiple effects of CFTR including ion transport, anti-inflammation, and anti-fibrosis were observed in the early stage of CS exposure, these effects ultimately contributed to the reversal of COPD development after chronic CS exposure. With the prolongation of CS exposure time, mice lacking CFTR developed alveolar remodeling similar to emphysema [3, 6]. In our study, genetic deletion of *SphK2* also contributed to emphysema-like changes following chronic CS exposure. However, the potential role of SphK2 in regulating the CFTR function and CS-induced pulmonary fibrosis and emphysema remained unclear. Notably, in recent studies, S1P was considered as a novel repressor of CFTR [16]. Cystic fibrosis caused by CFTR mutation is closely related to the development of COPD [2, 3], and abnormal S1P signaling was reported in a CFTR^F508del^ mutant mouse model of cystic fibrosis. In this study, we found that CFTR protein expression was significantly increased after SphK2 inhibition. However, administration of S1P_1_ agonist in *SphK2*^−/−^ mice markedly suppressed CFTR expression, abolished the protective effect of SphK2 deficiency on small airways remodeling and pulmonary inflammation after CS exposure. Our data strongly suggest that functional regulation of CFTR was critically involved in SphK2/S1P signaling-mediated pulmonary fibrosis and inflammation, and CFTR dysfunction has a close link to SphK2/S1P activation-mediated airways remodeling and emphysema after long-term CS exposure.

In addition to the inhibition of CFTR, Nf-κB activity was significantly upregulated in lung tissue after CS exposure [44, 45]. Nf-κB activation is strongly associated with CS-induced pulmonary inflammation, which aggravates apoptosis, autophagy, and remodeling of alveolar endothelial and epithelial cells [45–47]. Abnormal S1P level is a proinflammatory factor regulating endothelial barrier and vascular integrity [15, 16]. Here, we further established that S1P promoted CS-induced pulmonary inflammation by suppressing CFTR expression in alveolar epithelium, which might impair the barrier integrity and local inflammatory exudation. Indeed, we detected a high proportion of CD45^+^CD11b^+^ presenting leukocytes, and high levels of proinflammatory cytokines in the BALF collected from WT mice. The CD45^+^CD11b^+^ subpopulation is generally accepted as neutrophils accumulating at sites of injury after acute pulmonary inflammation [36]. We found these cells might contribute to CS-mediated inflammation by promoting the secretion of IL-6 and IL-33. This subpopulation could further divide into F4/80^+^ macrophages and Gr1^+^ monocytes. However, CS-induced pulmonary inflammation was largely improved in *SphK2*^−/−^ mice, both the CD45^+^CD11b^+^ subpopulation and the production of IL-6 and IL-33 decreased, indicating that SphK2 deficiency attenuated pulmonary inflammation and small airways fibrosis, and delayed emphysema pathogenesis possibly by reducing S1P production, blunting the S1P signaling and preserving the pulmonary CFTR function.

**Figure 6.**
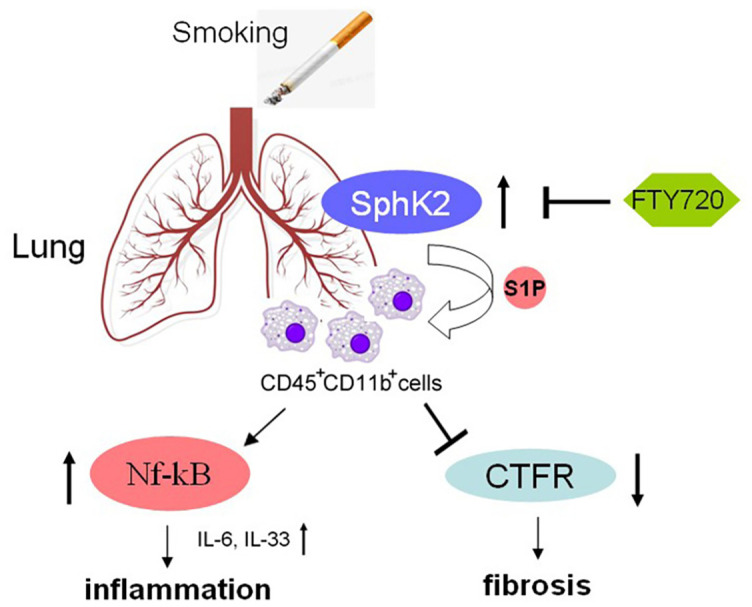
**Schematic diagram showing signaling pathways involved in SphK2-induced pulmonary inflammation and fibrosis after chronic CS exposure.** SphK2: Sphingosine kinase 2; CS: Cigarette smoke; WT: Wild-type; CFTR: cystic fibrosis transmembrane conductance regulator; IL: Interleukin.

To further confirm the inhibitory effect of S1P on CFTR activity, we pretreated the CS-exposed mice with FTY720, an S1P analog, which competes with S1P to bind with S1P receptor [16, 32]. FTY720 is an oral immunosuppressant which is used as an anti-asthmatic drug by inhibiting ceramide biosynthesis [32, 48]. Recently, the anti-inflammatory effect of FTY720 in acute lung injury model has also been widely reported. For example, FTY720 significantly decreases exudate due to lung vascular leak and inflammation [49], and FTY720 attenuated lung ischemia-reperfusion injury by reducing vascular permeability and the expression of proinflammatory cytokines such as IL-6, IL-17, and tumor necrosis factor α [50, 51]. Consistent with the previous reports, we found that FTY720 pretreatment significantly attenuated CS-mediated pulmonary accumulation of CD45^+^CD11b^+^ neutrophils and suppressed the secretion of IL-6 and IL-33. Apart from the anti-inflammatory effects, treatment with FTY720 also markedly inhibited lung fibrosis by downregulating the expression of α-SMA, antagonizing transforming growth factor β signaling and reversing the EMT progress [50]. Consistently, we found that collagen deposition in small airways induced by CS was significantly suppressed by FTY720 treatment. Notably, FTY720 also inhibited the phosphorylation level of Nf-κB-p65 and rescued the CFTR activity [52]. Therefore, regulation of pulmonary immune microenvironment by antagonizing S1P might contribute to alleviation of inflammation and fibrosis after chronic CS exposure. By contrast, the opposite effect of FTY720 on pulmonary fibrosis was also reported [16]. FTY720 aggravated lung injury and promoted fibrosis when administered during the remodeling phase [16]. These contrasting results suggest, once again, that S1P level is strongly associated with pulmonary endothelial and epithelial barrier, which is inconsistent with the results of CS-induced experimental COPD and other lung injury models [16]. Here, CS-induced hyperactive S1P signaling resulted in increased vascular permeability and infiltration of CD45^+^CD11b^+^ monocytes, which enhanced pulmonary inflammation, fibrosis, and remodeling (Figure 6). Such a pathophysiology was alleviated in CS-exposed *SphK2*^−/−^ mice, possibly due to the reduced synthesis of S1P. A similar effect was observed during early administration of FTY720, which also prevented pulmonary vascular and small airways dysfunction induced by CS by restoring S1P signaling to physiological baseline levels [53, 54].

### Study limitation

Mice model induced by CS is one of the classic animal models of emphysema. Although this model has been widely used in the studies related to molecular mechanisms of COPD previously, it is not exactly the same as human COPD symptoms. Human COPD is characterized by coughing, wheezing, chest tightness and shortness of breath, which are not observed in our CS-induced mice model. In addition, airway inflammation and pulmonary fibrosis are not typical symptoms in COPD patients, while lung infiltration with inflammatory cells and enhanced fibrosis were observed in mice exposed to CS for two months. In fact, COPD symptoms do not appear until severe lung injury occurs, usually lasting for years and worsening over time. In our study, CS-mediated mice model mimics the early stage of the pathological changes of COPD. Pulmonary inflammation and fibrosis may persist even after smoking cessation, but both will be attenuated during the bronchus and alveolar remodeling. Therefore, our observation indicates that the therapeutic intervention of SphK2/S1P signaling pathway may be effective in the early treatment of emphysema but may not be applicable to the research and treatment of end-stage COPD.

## Conclusion

Our study demonstrated a crucial role of SphK2 in the pathogenesis of COPD-like disease in CS-exposed mice. SphK2 deficiency reduced S1P production and therefore rescued CFTR activity and preserved pulmonary vascular function, small airways function, and endothelial/epithelial integrity. Hyperactivation of S1P signaling aggravated pulmonary inflammation by increasing the infiltration of CD45^+^CD11b^+^ monocytes and enhancing pulmonary fibrosis and remodeling, which are strongly linked to the development of COPD. Further studies are needed to elucidate the molecular mechanism of CFTR dysfunction due to SphK2 activation. Our findings highlight the role of S1P signaling inhibition as a therapeutic intervention to control the pathogenesis of emphysema in COPD.

## Supplemental Data
